# Supplementary figures and images for: Laboratory-based versus population-based surveillance of antimicrobial resistance to inform empirical treatment for suspected urinary tract infection in Indonesia
Source: PLoS One. 2020 Mar 30;15(3):e0230489. doi: 10.1371/journal.pone.0230489 (PMC7105116; doi:10.1371/journal.pone.0230489)

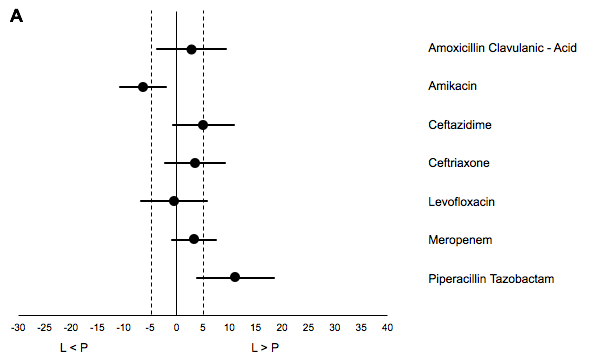


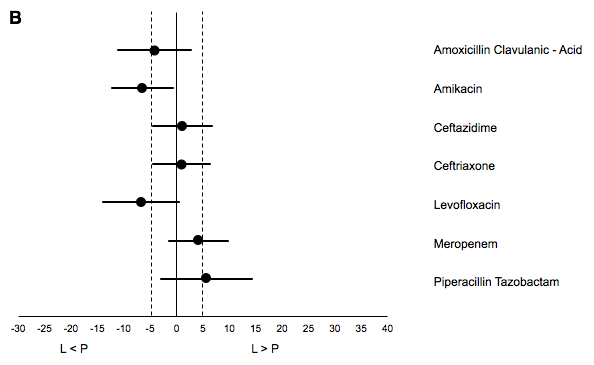


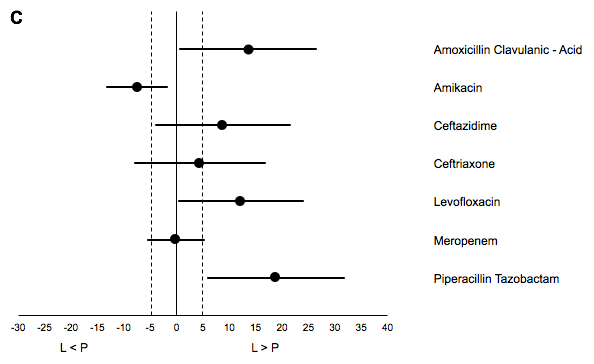


**S1 Fig**.

Supplement: S1 Fig — (A) Total (inpatient & outpatient setting) (B) Inpatients; (C) Outpatients; L>P = Laboratory-based surveillance prevalence estimate of resistance higher than population-based surveillance estimate. Bullets: percentage difference between laboratory- and population-based surveillance. Horizontal lines: confidence interval for the difference between the two prevalence estimates. Vertical dotted lines indicate to definition of bias (+/-5 percent point difference in prevalence estimate). (DOCX) [file pone.0230489.s001.docx]
