# Supplementary material for: Laboratory-based versus population-based surveillance of antimicrobial resistance to inform empirical treatment for suspected urinary tract infection in Indonesia
Source: PLoS One. 2020 Mar 30;15(3):e0230489. doi: 10.1371/journal.pone.0230489 (PMC7105116; doi:10.1371/journal.pone.0230489)
Supplement: S1 Table — Abbrev: n, number of isolates; R, number of resistance isolates; %R, resistance percentage; L, Laboratory-based data; P, Population-based data; %D, Percentage point difference; B, Bias; Y, Yes; N, No; CI, Confidence Interval; lb, lower boundaries; ub, upper boundaries; AMC, Amoxicillin Clavulanic–Acid; AK, Amikacin; CAZ, Ceftazidime; CRO, Ceftriaxone; LVX, Levofloxacin; MEM, Meropenem; TZP, Piperacillin Tazobactam. (DOCX) [file pone.0230489.s002.docx]

**S1 Table.**

| Antimicrobial  Drugs | L | | | P | | | %D | 95% CI | |
| --- | --- | --- | --- | --- | --- | --- | --- | --- | --- |
|  | n | R | %R | n | R | %R | L-P | lb | ub |
| AMC | 235 | 184 | 78.3 | 508 | 322 | 63.4 | 14.9 | 8.2 | 21.6 |
| AK | 439 | 28 | 6.4 | 508 | 50 | 9.8 | -3.5 | -6.9 | 0 |
| CAZ | 439 | 376 | 85.7 | 508 | 366 | 72.1 | 13.6 | 8.5 | 18.7 |
| CRO | 439 | 378 | 86.1 | 508 | 377 | 74.2 | 11.9 | 6.9 | 16.9 |
| LVX | 381 | 316 | 82.9 | 508 | 354 | 69.7 | 13.3 | 7.8 | 18.8 |
| MEM | 439 | 48 | 10.9 | 508 | 30 | 5.9 | 5.0 | 1.5 | 8.6 |
| TZP | 196 | 121 | 61.7 | 508 | 210 | 41.3 | 20.4 | 12.4 | 28.4 |
