# Supplementary material for: Laboratory-based versus population-based surveillance of antimicrobial resistance to inform empirical treatment for suspected urinary tract infection in Indonesia
Source: PLoS One. 2020 Mar 30;15(3):e0230489. doi: 10.1371/journal.pone.0230489 (PMC7105116; doi:10.1371/journal.pone.0230489)
Supplement: S2 Table — Abbrev: n, number of isolates; R, number of resistance isolates; %R, resistance percentage; L, Laboratory-based data; P, Population-based data; %D, Percentage point difference; B, Bias; Y, Yes; N, No; CI, Confidence Interval; lb, lower boundaries; ub, upper boundaries; AMC, Amoxicillin Clavulanic–Acid; AK, Amikacin; CAZ, Ceftazidime; CRO, Ceftriaxone; LVX, Levofloxacin; MEM, Meropenem; TZP, Piperacillin Tazobactam. (DOCX) [file pone.0230489.s003.docx]

**S2 Table.**

| Antimicrobial  Drugs | L | | | P | | | %D | 95% CI | |
| --- | --- | --- | --- | --- | --- | --- | --- | --- | --- |
|  | n | R | %R | n | R | %R | L-P | lb | ub |
| AMC | 163 | 137 | 84.1 | 247 | 202 | 81.8 | 2.3 | -5.1 | 9.7 |
| AK | 293 | 26 | 8.9 | 247 | 35 | 14.2 | -5.3 | -10.7 | 0.1 |
| CAZ | 293 | 274 | 93.5 | 247 | 221 | 89.6 | 4.0 | -0.7 | 8.8 |
| CRO | 293 | 275 | 93.9 | 247 | 225 | 91.1 | 2.8 | -1.7 | 7.3 |
| LVX | 258 | 210 | 81.4 | 247 | 202 | 81.8 | -0.4 | -7.1 | 6.4 |
| MEM | 293 | 43 | 14.7 | 247 | 20 | 8.1 | 6.6 | 1.3 | 11.9 |
| TZP | 130 | 92 | 70.8 | 247 | 141 | 57.1 | 13.7 | 3.7 | 23.6 |
