# Supplementary material for: Laboratory-based versus population-based surveillance of antimicrobial resistance to inform empirical treatment for suspected urinary tract infection in Indonesia
Source: PLoS One. 2020 Mar 30;15(3):e0230489. doi: 10.1371/journal.pone.0230489 (PMC7105116; doi:10.1371/journal.pone.0230489)
Supplement: S3 Table — Abbrev: n, number of isolates; R, number of resistance isolates; %R, resistance percentage; L, Laboratory-based data; P, Population-based data; %D, Percentage point difference; B, Bias; Y, Yes; N, No; CI, Confidence Interval; lb, lower boundaries; ub, upper boundaries; AMC, Amoxicillin Clavulanic–Acid; AK, Amikacin; CAZ, Ceftazidime; CRO, Ceftriaxone; LVX, Levofloxacin; MEM, Meropenem; TZP, Piperacillin Tazobactam. (DOCX) [file pone.0230489.s004.docx]

**S3 Table.**

| Antimicrobial  Drugs | L | | | P | | | %D | 95% CI | |
| --- | --- | --- | --- | --- | --- | --- | --- | --- | --- |
|  | n | R | %R | n | R | %R | L-P | lb | ub |
| AMC | 72 | 47 | 65.3 | 261 | 120 | 46.0 | 19.3 | 6.8 | 31.9 |
| AK | 146 | 2 | 1.4 | 261 | 15 | 5.8 | -4.4 | -7.8 | -1.0 |
| CAZ | 146 | 102 | 69.9 | 261 | 145 | 55.6 | 14.3 | 4.7 | 23.9 |
| CRO | 146 | 103 | 70.6 | 261 | 152 | 58.2 | 12.3 | 2.8 | 21.8 |
| LVX | 123 | 106 | 86.2 | 261 | 152 | 58.2 | 27.9 | 19.4 | 36.5 |
| MEM | 146 | 5 | 3.4 | 261 | 10 | 3.8 | -0.4 | -4.2 | 3.4 |
| TZP | 66 | 29 | 43.9 | 261 | 69 | 26.4 | 17.5 | 4.4 | 30.6 |
