# Supplementary material for: Laboratory-based versus population-based surveillance of antimicrobial resistance to inform empirical treatment for suspected urinary tract infection in Indonesia
Source: PLoS One. 2020 Mar 30;15(3):e0230489. doi: 10.1371/journal.pone.0230489 (PMC7105116; doi:10.1371/journal.pone.0230489)
Supplement: S4 Table — Abbrev: n, number of isolates; R, number of resistance isolates; %R, resistance percentage; L, Laboratory-based data; P, Population-based data; %D, Percentage point difference; B, Bias; Y, Yes; N, No; CI, Confidence Interval; lb, lower boundaries; ub, upper boundaries; AMC, Amoxicillin Clavulanic–Acid; AK, Amikacin; CAZ, Ceftazidime; CRO, Ceftriaxone; LVX, Levofloxacin; MEM, Meropenem; TZP, Piperacillin Tazobactam. (DOCX) [file pone.0230489.s005.docx]

**S4 Table.**

| Antimicrobial  Drugs | L | | | P | | | %D | 95% CI | |
| --- | --- | --- | --- | --- | --- | --- | --- | --- | --- |
|  | n | R | %R | n | R | %R | L-P | lb | ub |
| AMC | 235 | 184 | 78.3 | 281 | 205 | 73.0 | 5.3 | -2.1 | 12.7 |
| AK | 439 | 28 | 6.4 | 281 | 34 | 12.1 | -5.7 | -10.2 | -1.3 |
| CAZ | 439 | 376 | 85.6 | 281 | 223 | 79.4 | 6.3 | 0.5 | 12.0 |
| CRO | 439 | 378 | 86.1 | 281 | 229 | 81.5 | 4.6 | -1.0 | 10.2 |
| LVX | 381 | 316 | 82.9 | 281 | 217 | 77.2 | 5.7 | -0.5 | 11.9 |
| MEM | 439 | 48 | 10.9 | 281 | 21 | 7.5 | 3.5 | -0.8 | 7.7 |
| TZP | 196 | 121 | 61.7 | 281 | 146 | 52.0 | 9.8 | 0.8 | 18.7 |
