# Supplementary material for: Laboratory-based versus population-based surveillance of antimicrobial resistance to inform empirical treatment for suspected urinary tract infection in Indonesia
Source: PLoS One. 2020 Mar 30;15(3):e0230489. doi: 10.1371/journal.pone.0230489 (PMC7105116; doi:10.1371/journal.pone.0230489)
Supplement: S5 Table — Abbrev: n, number of isolates; R, number of resistance isolates; %R, resistance percentage; L, Laboratory-based data; P, Population-based data; %D, Percentage point difference; B, Bias; Y, Yes; N, No; CI, Confidence Interval; lb, lower boundaries; ub, upper boundaries; AMC, Amoxicillin Clavulanic–Acid; AK, Amikacin; CAZ, Ceftazidime; CRO, Ceftriaxone; LVX, Levofloxacin; MEM, Meropenem; TZP, Piperacillin Tazobactam. (DOCX) [file pone.0230489.s006.docx]

**S5 Table.**

| Antimicrobial  Drugs | L | | | P | | | %D | 95% CI | |
| --- | --- | --- | --- | --- | --- | --- | --- | --- | --- |
|  | n | R | %R | n | R | %R | L-P | lb | ub |
| AMC | 163 | 137 | 84.0 | 184 | 153 | 83.2 | 0.9 | -6.9 | 8.7 |
| AK | 293 | 26 | 8.9 | 184 | 26 | 14.1 | -5.3 | -11.3 | 0.7 |
| CAZ | 293 | 274 | 93.5 | 184 | 164 | 89.1 | 4.4 | -0.9 | 9.7 |
| CRO | 293 | 275 | 93.9 | 184 | 165 | 89.7 | 4.2 | -1.0 | 9.4 |
| LVX | 258 | 210 | 81.4 | 184 | 152 | 82.6 | -1.2 | -8.5 | 6.0 |
| MEM | 293 | 43 | 14.7 | 184 | 17 | 9.2 | 5.4 | -0.4 | 11.3 |
| TZP | 130 | 92 | 70.8 | 184 | 113 | 61.4 | 9.4 | -1.2 | 19.9 |
