# Supplementary material for: Laboratory-based versus population-based surveillance of antimicrobial resistance to inform empirical treatment for suspected urinary tract infection in Indonesia
Source: PLoS One. 2020 Mar 30;15(3):e0230489. doi: 10.1371/journal.pone.0230489 (PMC7105116; doi:10.1371/journal.pone.0230489)
Supplement: S6 Table — Abbrev: n, number of isolates; R, number of resistance isolates; %R, resistance percentage; L, Laboratory-based data; P, Population-based data; %D, Percentage point difference; B, Bias; Y, Yes; N, No; CI, Confidence Interval; lb, lower boundaries; ub, upper boundaries; AMC, Amoxicillin Clavulanic–Acid; AK, Amikacin; CAZ, Ceftazidime; CRO, Ceftriaxone; LVX, Levofloxacin; MEM, Meropenem; TZP, Piperacillin Tazobactam. (DOCX) [file pone.0230489.s007.docx]

**S6 Table.**

| Antimicrobial  Drugs | L | | | P | | | %D | 95% CI | |
| --- | --- | --- | --- | --- | --- | --- | --- | --- | --- |
|  | n | R | %R | n | R | %R | L-P | lb | ub |
| AMC | 72 | 47 | 65.3 | 97 | 52 | 53.6 | 11.7 | -3.1 | 26.5 |
| AK | 146 | 2 | 1.4 | 97 | 8 | 8.3 | -6.9 | -12.7 | -1.1 |
| CAZ | 146 | 102 | 69.9 | 97 | 59 | 60.8 | 9.0 | -3.2 | 21.3 |
| CRO | 146 | 103 | 70.5 | 97 | 64 | 66.0 | 4.6 | -7.4 | 16.6 |
| LVX | 123 | 106 | 86.2 | 97 | 65 | 67.0 | 19.2 | 8.0 | 30.3 |
| MEM | 146 | 5 | 3.4 | 97 | 4 | 4.1 | -0.7 | -5.6 | 4.2 |
| TZP | 66 | 29 | 43.9 | 97 | 33 | 34.0 | 9.9 | -5.3 | 25.2 |
